# Insights into Thermal Interactions in Frozen Pharmaceutical Vials: Effects on Ice Nucleation Times and Inhibition

**DOI:** 10.1007/s11095-024-03713-2

**Published:** 2024-05-20

**Authors:** Roberto Pisano, Jessica Semeraro, Fiora Artusio, Antonello A. Barresi

**Affiliations:** Department of Applied Science and Technology, Politecnico di Torino, 24 Corso Duca Degli Abruzzi, IT10129 Turin, Italy

**Keywords:** batch heterogeneity, drug products, freezing, thermal interactions

## Abstract

**Purpose:**

This study investigates the thermal interactions between adjacent vials during freezing and assesses their impact on nucleation times.

**Methods:**

Various loading configurations were analyzed to understand their impact on nucleation times. Configurations involving direct contact between vials and freeze-dryer shelves were studied, along with setups using empty vials between filled ones. Additionally, non-conventional loading configurations and glycol-filled vials were tested. The analysis includes 2R and 20R vials, which are commonly utilized in the freezing and lyophilization of drug products, along with two different fill depths, 1 and 1.4 cm.

**Results:**

The investigation revealed that configurations with direct contact between vials and freeze-dryer shelves led to substantial thermal interactions, resulting in delayed nucleation in adjacent vials and affecting the temperature at which nucleation takes place in a complex way. In another setup, empty vials were placed between filled vials, significantly reducing thermal interactions. Further tests with non-conventional configurations and glycol-filled vials confirmed the presence of thermal interactions with a minimal inhibitory effect.

**Conclusions:**

These findings carry significant implications for the pharmaceutical industry, highlighting the role of thermal interactions among vials during freezing and their impact on the temperature at which ice nucleation occurs.

## Introduction

The storage and distribution of biopharmaceuticals have become a topic of increasing concern, with particular attention to the challenges of maintaining specific temperature conditions. Most biopharmaceuticals necessitate cold storage, including some lyophilized drug products, typically between 2 to 8 °C, or ultra-cold storage at temperatures of –20 or –70 °C.

Several considerations come into play when it comes to products stored in a frozen state or subjected to lyophilization. Freezing can compromise the therapeutic effectiveness of the drug formulation. For instance, low temperatures can lead to denaturation of therapeutic proteins due to reduced water-hydrophobic interactions [1–5]. Moreover, the formation of the ice-water interface can induce changes in the native protein fold [6, 7] and potentially accelerate chemical reactions through alterations in ionic strength and amorphous phase composition. Variations in pH, phase separation of polymers, and selective crystallization of excipients can further impact protein activity, making the freezing process a critical factor in product stability [8–10]. Therefore, the choice of freezing conditions becomes pivotal in either preventing or promoting these undesired occurrences, and the precise design of the freezing process is essential within the pharmaceutical industry [11].

The freezing process involves two distinct phases; a cooling phase, where the solution remains in a liquid state, and a solidification phase, characterized by ice formation. The latter is initiated by a stochastic process, known as ice nucleation, which introduces variability in freezing behavior among vials, even when their thermal environment is identical [12–19]. Unfortunately, even minor differences in nucleation time and temperature can lead to variations in ice crystal size, which plays a pivotal role in maintaining protein stability, with larger ice crystals being more favorable.

When dealing with drug products vulnerable to freezing-induced degradation, it is imperative to consider three parameters: nucleation time, nucleation temperature, and solidification time. The nucleation time defines the duration during which the solution remains in a liquid state under supercooled conditions, rendering it susceptible to cold denaturation. The nucleation temperature is the primary determinant of ice crystal shape and size and, consequently, of the extent of the ice-water surface area [20]. Lastly, the solidification time is tightly related to the cooling rate and defines the duration of the freeze-concentration phase [21–23] and can further influence the ice crystal size. When the cooling rate is high, such as rapid freezing with liquid nitrogen, the formation of smaller ice crystals is favored [24, 25]. However, in industrial practice, the cooling rate typically falls within a range of 0.1 to 1 °C min^−1^, and the nucleation temperature becomes the primary determinant of ice crystal size. Higher nucleation temperatures lead to the formation of larger ice crystals [26].

Unfortunately, precisely controlling the nucleation temperature is challenging, as it exhibits a stochastic distribution within a batch of vials, making it unpredictable [13, 27]. Attempts have been made to develop technologies that can induce ice nucleation within a narrower temperature range [12]; however, these solutions are still in the experimental stage and are rarely integrated into manufacturing processes [28, 29]. The unpredictability of ice nucleation leads to variations in product morphology, residual biological activity, and, in the case of lyophilization, residual moisture and reconstitution time. These differences in product quality can impact the critical attributes of frozen and lyophilized drug products, emphasizing the need to consider batch heterogeneity when designing the freezing process [13, 14].

The potential impact of freezing conditions on the freeze-drying process, and on the quality attributes of the drug product, has motivated various studies in recent times. Many researchers have turned to mathematical modeling to predict the distribution of ice nucleation temperatures among vials nested in pallets and have linked this distribution to frozen product morphology [16, 17, 30–32]. Moreover, the heat released by a nucleated vial can potentially delay ice nucleation in neighboring vials, further complicating the freezing process and varying the heat transfer efficiency between adjacent vials, which depends on the loading configuration [17]. While previous studies have explored the influence of packing density and loading configuration on batch uniformity, they have predominantly focused on heat transfer during primary drying [33–36].

Pisano *et al*. [27] have recently compared two loading configurations, vials directly resting on the shelf and nested in a rack system. They found that nucleation time distributions change when a customized holder separates vials from one another and lifts them off the refrigerated shelves. This loading configuration minimizes the potential for thermal interactions among neighboring vials while concomitantly reducing the cooling rates.

On a commercial scale, where tens of thousands of vials are commonly grouped within pallets and subjected to slow freezing within cold storage facilities, the interplay of stochastic ice nucleation and spatial variations in heat transfer complicates the process [16, 17]. The specific configuration of densely packed vials significantly affects freezing behavior, as thermal interactions among neighboring vials are unavoidable. The same issue arises in batches of vials subjected to freezing before undergoing lyophilization.

In conclusion, many studies have elucidated the correlation between frozen product characteristics, such as mean ice crystal size, and the freezing conditions. However, despite their valuable contributions, these insights have not yet impacted the redesign of the freezing process in the industry. One of the primary reasons for this limited impact is the predominant focus of these studies on the single-vial scale. To bridge the existing research at the individual (and isolated) vial scale with the practical process design on a batch scale, i.e., hundreds of vials in contact one another, this study addresses the influence of thermal interactions between neighboring vials on nucleation time distribution within a batch.

## Materials and Methods

### Materials

Freezing experiments were conducted using 4 cc tubing vials (2R ISO, Soffieria Bertolini, Candiolo, Italy) filled with 2 mL of a 5 wt% sucrose solution (corresponding to 1.4 cm as fill height) or 50 vol% ethylene glycol solution. 2R vials were used to allow the visualization of a matrix of 10 × 20 vials through video cameras, guaranteeing statistical significance of the dataset. Additional experiments were also performed in 20R vials filled with 5 mL (corresponding to 1 cm as fill height) of a 5 wt% sucrose solution. Vials were left unstoppered to facilitate the monitoring of ice nucleation using video cameras. The sucrose and ethylene glycol, sourced from Merck, were used as-is without additional purification, and the solutions were prepared using water for injection (Fresenius Kabi, Milan, Italy). All reagents used in these experiments were of analytical grade, and the resulting solutions were filtered through a 0.2 µm syringe filter (PVDF, Merck, Milan, Italy) before use.

### Experimental Setup

**Fig. 1:**
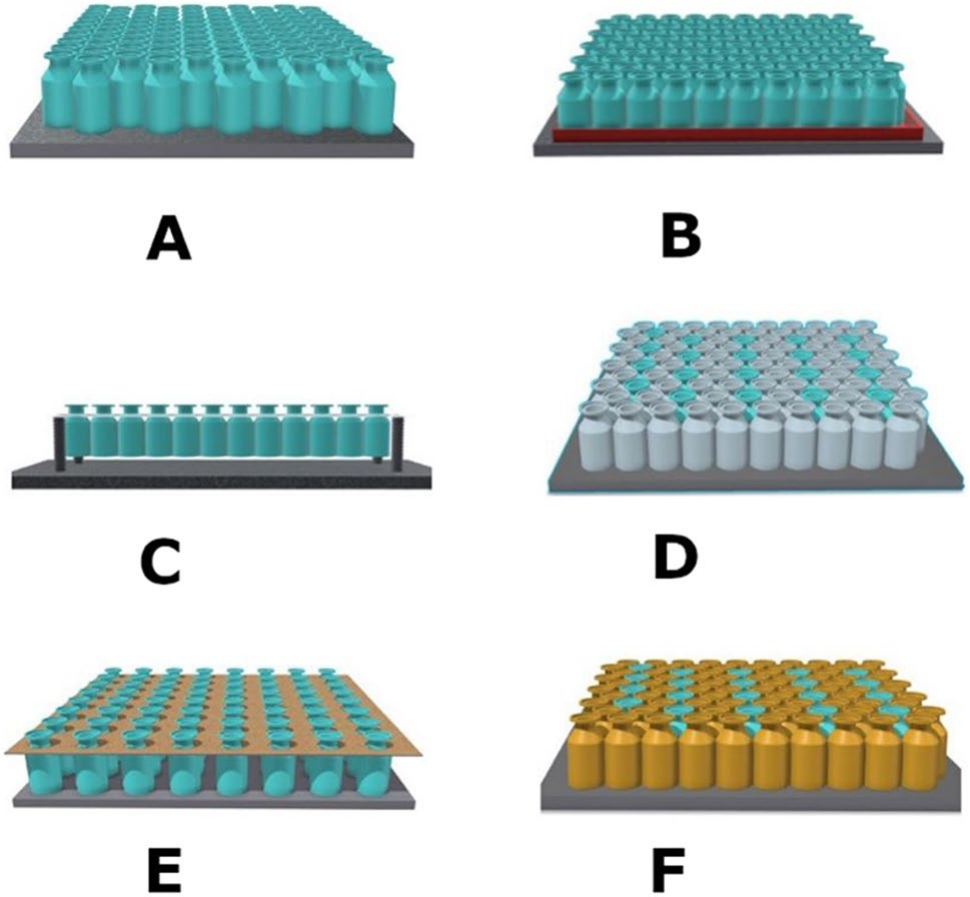
Schematic of the various loading configurations for vials used in this study. Various colors were used to identify vials containing  sucrose,  ethylene glycol, and  empty vials.

The freezing runs were conducted using various loading configurations in a lab-scale freeze-dryer (Revo, Millrock Technology, Kingston, New York, USA). These configurations aim to explore potential thermal interactions during freezing and evaluate whether such interactions might result in changes in the distribution of nucleation times. The various configurations are depicted in Fig. 1 and include:

[A] Vials are in contact with one another and resting on the refrigerated shelf. Each vial is filled with 2 mL of a 5 wt% sucrose solution. They are arranged in a hexagonal pattern, so each vial is in contact with six neighboring vials. An additional test was performed using 20R vials filled with 5 mL of a 5 wt% sucrose solution loaded in a hexagonal configuration.
[B] Vials are in contact with one another and loaded on a stainless-steel tray placed on the refrigerated shelf. Each vial contains 2 mL of a 5 wt% sucrose solution. Similar to configuration A, vials are arranged in a hexagonal pattern, and each vial is in contact with six neighboring vials.
[C] Vials are loaded onto a support that suspends them above the shelf. In this configuration, the vials are arranged in a single line, so each vial is in contact with two neighboring vials.
[D] Vials are in contact with one another and resting on the refrigerated shelf. Some vials are filled with 2 mL of a 5 wt% sucrose solution. Six empty vials surrounded each filled vial.
[E] Vials are resting on the shelf, and a customized holed spacer separates them from one another. The empty space between neighboring vials is approximately 6 mm.
[F] Similar to configuration D, empty vials are substituted by vials containing 2 mL of ethylene glycol solution to delay nucleation and quantify the thermal interaction caused by the nucleation of the center sucrose vial. The ethylene glycol solution has a freezing point equal to —36 °C, thus ensuring that these vials nucleate after all the vials filled with sucrose solution.

Table I summarizes the characteristics of the various configurations in terms of batch size and composition, and the type of contact between the vials and the refrigerated shelves.

For each configuration, we performed three freeze–thaw cycles. During these cycles, the vials were cooled to –45 °C at 0.5 °C min^–1^, held at that temperature for 20 min, and then gradually thawed at 3 °C min^–1^ to + 25 °C. The shelf temperature was kept at –45 °C for the shortest time possible to minimize ice formation outside the vials due to humidity in the chamber.

**Table I:** Experimental setup used for the various freezing runs. ${N}_{v,tot}$ is the total number of vials, ${N}_{v,s}$ is the number of vials containing sucrose solution, ${N}_{v,empty}$ is the number of empty vials, and ${N}_{v,gly}$ is the number of vials containing ethylene glycol solution

Configuration	${N}_{v,tot}$	${N}_{v,s}$	${N}_{v,empty}$	${N}_{v,gly}$	Shelf contact	Tray	Vial contact
A	196	196	–	–	Yes	No	Yes
B	196	196	–	–	Yes	Yes	Yes
C	40	40	–	–	No	No	Yes
D	196	36	160	–	Yes	No	Yes
E	100	100	–	–	Yes	No	No
F	196	36	–	160	Yes	No	Yes

Two video cameras recorded the freezing process in all experimental configurations at a frame rate of 5 fps. These cameras were strategically positioned on opposite sides of the temperature-controlled shelves to monitor the freezing behavior of a matrix consisting of 10 × 20 vials.

**Fig. 2:**
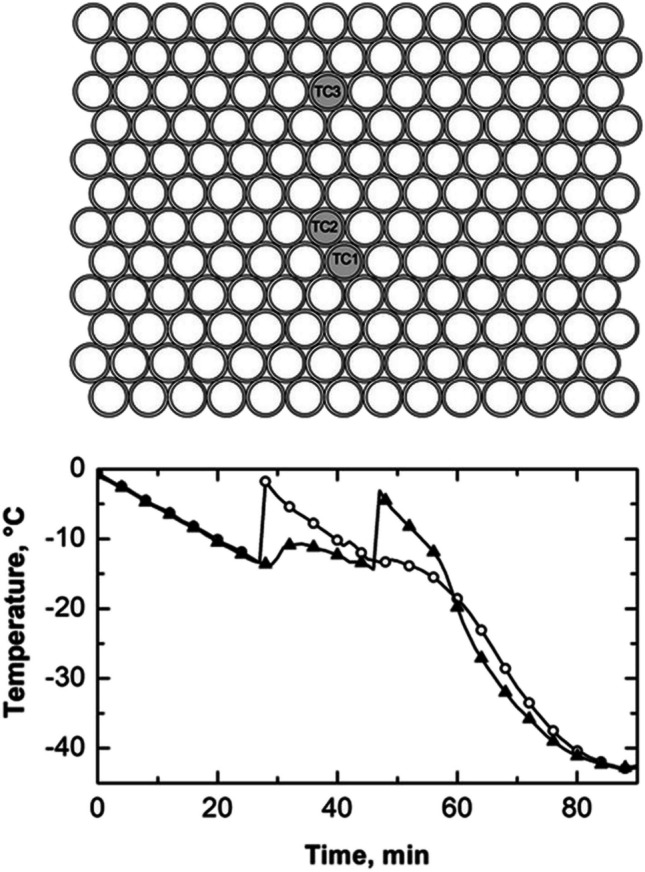
(top) Schematic illustrating the positioning of three thermocouples within the batch when vials are in direct contact with the freeze-dryer shelves (configuration A). (bottom) Temperature profiles of thermocouples TC1 (vial#1, ○) and TC2 (vial#2, ▲). The initial time was fixed at the time instant when the three thermocouples reached 0 °C.

The ice nucleation time of individual vials (${t}_{n}$) was determined as the time instant at which the solution becomes visibly cloudy. This parameter is defined as the time elapsed from the point at which the temperature of a specific reference vial achieved 0 °C. The temperature was measured through three T-type miniature thermocouples (Tersid, Milan, Italy) placed in close proximity to the bottom of the vial and in strategic positions of the vial batch (see, for example, Fig. 2). The full measurement chain, from the sensor to the reader, was calibrated against a traceable Pt100 sensor. After the calibration, comparison tests carried out placing the thermocouple in ice showed a temperature error below 1 °C. It is worth noting that the detected time may exhibit slight variations from the actual nucleation time. This discrepancy arises from the fact that critical nuclei, being at the nanoscale, cannot be visually observed at the moment of their formation during nucleation. It is only when these critical nuclei reach a sufficient size to occupy a substantial volume fraction of the suspension that the solution becomes turbid [37].

This analysis focused exclusively on center vials in order to minimize potential biases stemming from edge-vial effects. Furthermore, the reference vials were intentionally excluded from the analysis to prevent potential interference caused by the thermocouple tips with the ice nucleation process.

### Statistical Analysis

A spatial autocorrelation analysis was conducted to examine the potential correlation between the nucleation times of adjacent vials. Positive spatial autocorrelation is observed when neighboring vials share similar nucleation time values, while negative autocorrelation indicates differences among neighboring vials. In cases where the distribution of the variable is random, no spatial autocorrelation is evident. Among the various indices for assessing spatial autocorrelation, we used the local Moran's index $\left({I}_{i}\right)$ to explore these patterns and the global Moran's index $\left(I\right)$ to assess the overall characteristics of the entire batch [38, 39].

In calculating the Moran index, we employ a neighbor matrix (or weight matrix) sized $n\times n$ to portray the spatial configuration:

$$\mathbf{W}={\left[{w}_{ij}\right]}_{n\times n}$$

where the element ${w}_{ij}$ equals 1 if vial $i$ is adjacent to vial $j$; otherwise, it is null. The local Moran index, concerning the $i$-th vial, is defined as,

$${I}_{i}=\frac{{z}_{i}}{{m}_{2}}\sum_{j=1}^{n}{w}_{ij}{z}_{j}$$

where ${m}_{2}$ is the second-order moment of the normalized nucleation time vector ($\mathbf{z}$). This last vector is calculated from the measured nucleation vector ($\mathbf{x}$), whose mean value and standard deviation are,

$$\mu =\frac{1}{n}\sum_{i=1}^{n}{x}_{i}$$

$$\sigma =\sqrt{\frac{1}{n}\sum_{i=1}^{n}{\left({x}_{i}-\mu \right)}^{2}}$$

The normalized nucleation time $\mathbf{z}$ is,

$$\mathbf{z}=\frac{\mathbf{y}}{\sigma }$$

where $\mathbf{y}$ is the nucleation time vector centered around its mean value,

$$\mathbf{y}=\mathbf{x}-\mu \mathbf{i}$$

where $\mathbf{i}$ is a column vector of $n$ elements and values equal to 1.

Once the local Moran’s index has been calculated, its variance can be expressed as follows,

$${\sigma }_{I}=\frac{{w}_{i\left(2\right)}\left(n-{b}_{2}\right)}{n-1}+\frac{2{w}_{i\left(kh\right)}\left(2{b}_{2}-n\right)\left(n-2\right)}{\left(n-1\right)}-\frac{{w}_{i}^{2}}{{\left(n-1\right)}^{2}}$$

where ${w}_{i\left(2\right)}=\sum_{i\ne j}{w}_{ij}^{2}$, ${b}_{2}=\frac{\sum_{i}\left({~}^{{z}_{i}^{4}}\!\left/ \!{~}_{n}\right.\right)}{{\left({~}^{\sum_{i}{z}_{i}^{2}}\!\left/ \!{~}_{n}\right.\right)}^{2}}$, and $2{w}_{i\left(kh\right)}=\sum_{k\ne i}\sum_{h\ne i}{w}_{ik}{w}_{ih}$. It follows that the vector of centered and normalized local Moran's indices $\mathbf{Z}$ is,

$${Z}_{i}=\frac{{I}_{i}-{E}_{i}}{{\sigma }_{I}}$$

where ${E}_{i}=-\frac{{\sum }_{j=1}^{n}{w}_{ij}}{\left(n-1\right)}$. The global Moran's index is hence defined as,

$$I=\frac{\sum_{i=1}^{n}{I}_{i}}{\sum_{i=1}^{n}{w}_{i\left(2\right)}}$$

The results can be visualized in a scatter plot, where the centered and normalized nucleation time vector **z** is plotted on the x-axis and the centered and standardized vector of local Moran indices **Z** is plotted on the y-axis. A vial associated with a negative local Moran’s index indicates significant differences in nucleation times among its adjacent vials. Therefore, there is inhibition if most of the points reside in the third and fourth quadrants, where local Moran’s indices are negative.

## Results and Discussion

### Packed Vials Resting on the Shelf

In order to investigate thermal interactions between adjacent vials during the freezing process, vials were intentionally positioned in direct contact with both their neighboring vials and the freeze-dryer shelves. In this configuration, each vial was in contact with six neighboring vials, as depicted in Fig. 2.

All vials were filled with 2 mL of a 5 wt% sucrose solution, and the temperature of three vials was monitored by positioning a thermocouple at the bottom of each vial. Thermocouples TC1 and TC2 were strategically positioned to observe the influence of nucleation in one vial on the thermal behavior of the other. In addition, thermocouple TC3 was introduced to assess potential thermal gradients on the shelf surface.

As shown in Fig. 2 (bottom graph), during the first 25 min of the undercooling time, vials #1 and #2 cooled at a similar rate of about 0.5 °C min^–1^. Then, vial #1 nucleated, causing its temperature to reach the solution freezing point. The heat released during nucleation was partially transferred to vial #2, sharply reducing the cooling rate to 0.2 °C min^–1^. At the 48-min mark, the TC1 thermal profile showed another temperature increase, likely due to the nucleation of adjacent vials.

Vial #1 nucleated after approximately 25 min; its nucleation temperature was –12 °C, 2 °C higher than vial #2, even though vial #2 had been cooling for longer. This result suggests the existence of thermal interactions between adjacent vials during ice nucleation and crystallization. These interactions can delay the nucleation of neighboring vials, causing nucleation to occur at higher temperatures than expected, or it could change the expected distribution of nucleation temperatures when there are no thermal interactions. This phenomenon results from the heat released during nucleation and the growth of ice crystals in vials that have already nucleated. This result further demonstrates that it is impossible to establish a direct correlation between nucleation time and temperature. In other words, a vial that nucleates later does not necessarily do so at a lower temperature, even if there is uninterrupted cooling from the refrigerated shelf.

**Fig. 3:**
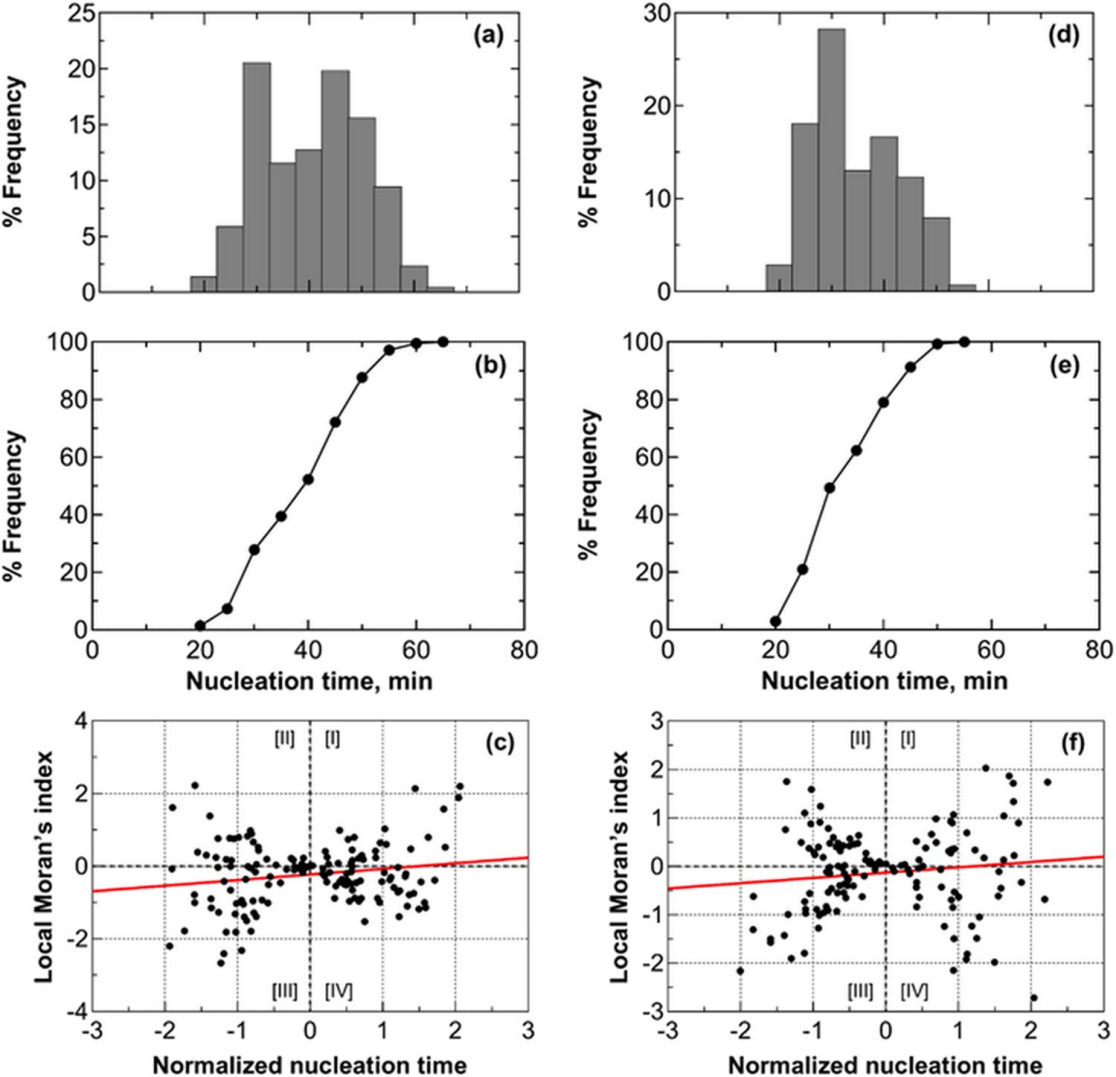
(**a**) Nucleation time distribution, (**b**) its cumulative function, and (**c**) scatterplot of standardized local Moran's indices for a batch of 2R vials in direct contact with one another and the freeze-dryer shelf (configuration A). Data refer to three replicas involving 196 vials, each filled with 2 mL of a 5 wt% sucrose solution. (**d**) Nucleation time distribution, (**e**) its cumulative function, and (**f**) scatterplot of standardized local Moran's indices for a batch of 20R vials in direct contact with one another and the freeze-dryer shelf (configuration A). Data refer to three replicas involving 49 vials, each filled with 5 mL of a 5 wt% sucrose solution. The vials with thermocouples were omitted from the statistical analysis to avoid any potential interference that the sensor probes might have on the ice nucleation process.

Figure 3a shows the evolution of the average distribution of nucleation times calculated from three replicas. Nucleation times exhibited a random distribution. On average, the time elapsed between the nucleation of the first vial in the batch and the nucleation of the last vial was 47 min. The distribution shows two peaks at 35 and 50 min. This bimodal distribution suggests an inhibitory effect due to the nucleation heat released by vials nucleating within the first peak on adjacent vials that have not yet nucleated. This hypothesis is supported when observing the behavior of thermocouples TC1 and TC2, as depicted in Fig. 2. Vial #1 nucleated within the first peak, while vial #2 belongs to the vials nucleating within the second peak.

In Fig. 3b, the cumulative distribution function reveals that after 35 min, 34% of the vials have nucleated. Between 35 and 50 min, an additional 47% of the vials underwent nucleation. It follows that after the second peak, 81% of the vials in the batch have achieved nucleation, while the remaining 19% experienced delayed nucleation due to the heat released by vials nucleating within the second peak.

In order to assess the consistency of the collected data, we compared the nucleation time distributions within a batch of vials that underwent three freeze–thaw cycles. Subsequently, we compared quartiles, standard deviation, and the range of nucleation times. As can be seen in Table II, these results provide strong evidence confirming the reproducibility of the test.

**Table II:** Statistical parameters of the nucleation time distribution curves for the three replicates of the freezing test on a batch of 2R vials, both in direct contact with one another and in contact with the freeze-dryer shelf

Replica	Mean, min	Median, min	Min, min	Max, min	IQR, min	Global Moran’s index, −
#1	42	43	19	66	16	–0.10
#2	41	42	20	62	16	–0.08
#3	40	41	21	60	15	–0.10

Figure 3c shows the locally centered Moran's indices, centered with respect to the expected value and normalized to standard deviation. Most data points fell within the third and fourth quadrants, where negative local Moran's indices confirm inhibition. A vial with a negative local Moran's index indicates significant variation in nucleation times among adjacent vials, explaining delayed nucleation in the center vial due to the surrounding six ones. The global Moran's indices, as presented in Table II, also affirm the presence of inhibition in all three test repetitions.

Additionally, the potential presence of thermal gradients over the shelf was assessed by comparing thermocouple readings at three crucial freezing phase time points: the onset of the cooling ramp (${t}_{in}$), the achievement of equilibrium freezing temperature (${t}_{0}$), and the completion of freezing (${t}_{f}$), i.e., when all the vials of the batch had nucleated.

**Table III:** Shelf temperature at three freezing phase time points and positions. TC1 and TC2 were inserted in vials placed in the central part of the shelf, while TC3 was close to the edge of the batch

**Vials loaded directly on the shelf (A)**									
Replica	#1			#2			#3		
TCs	TC1	TC2	TC3	TC1	TC2	TC3	TC1	TC2	TC3
${t}_{in}$	19.8	19.4	19.8	29.2	28.9	29.5	29.2	28.9	29.4
${t}_{0}$	1.0	–0.2	–0.2	0.9	–0.2	–0.2	1.4	–0.2	–0.2
${t}_{f}$	–43.6	–44.3	–43.7	–42.0	–42.8	–42.5	n.a	n.a	n.a
**Vials loaded on a metal tray (B)**									
Replica	#1			#2			#3		
TCs	TC1	TC2	TC3	TC1	TC2	TC3	TC1	TC2	TC3
${t}_{in}$	19.3	19.8	19.8	29.1	28.6	29.4	29.1	28.6	29.4
${t}_{0}$	0.8	0.8	–0.2	0.8	0.8	–0.2	0.3	0.3	–0.2
${t}_{f}$	–42.9	–42.6	–43	–	–	–	–	–	–

The thermal gradients observed on the surface of the shelf were a result of the path taken by the refrigerant fluid within the shelf. Vials near the refrigerant outlet were warmer than those near the inlet. Temperature variations also occurred between adjacent vials. At the start of freezing, thermocouples TC1 and TC2 had similar readings. However, when TC2 reached the equilibrium freezing temperature, TC1 acquired a higher temperature difference, ranging from 1.1 °C to 1.6 °C. TC3, positioned laterally, reached the same temperature as TC2. As freezing progresses to –45 °C, the gradient effects diminished, reducing the temperature difference between thermocouples to 0.7–0.8 °C.

An additional test was performed using a larger vial format, i.e., 20R, and a different fill height (1 cm). A similar behavior compared to the previous test performed on 2R vials was observed, as can be seen in Fig. 3d-f. The nucleation time distribution showed a first peak at 30 min, followed by a second wider peak. The cumulative distribution function reveals that after 35 min, 49% of the vials have nucleated. After 44 min, 88% of the vials have nucleated. The scatterplot of the local Moran’s indices highlights similar behavior to 2R vials, suggesting the presence of thermal interactions even in larger vials and smaller fill heights. Local Moran’s indices were more scattered compared to the test performed on 2R vials due to the smaller number of monitored vials. The global Moran's index was –0.06.

**Fig. 4:**
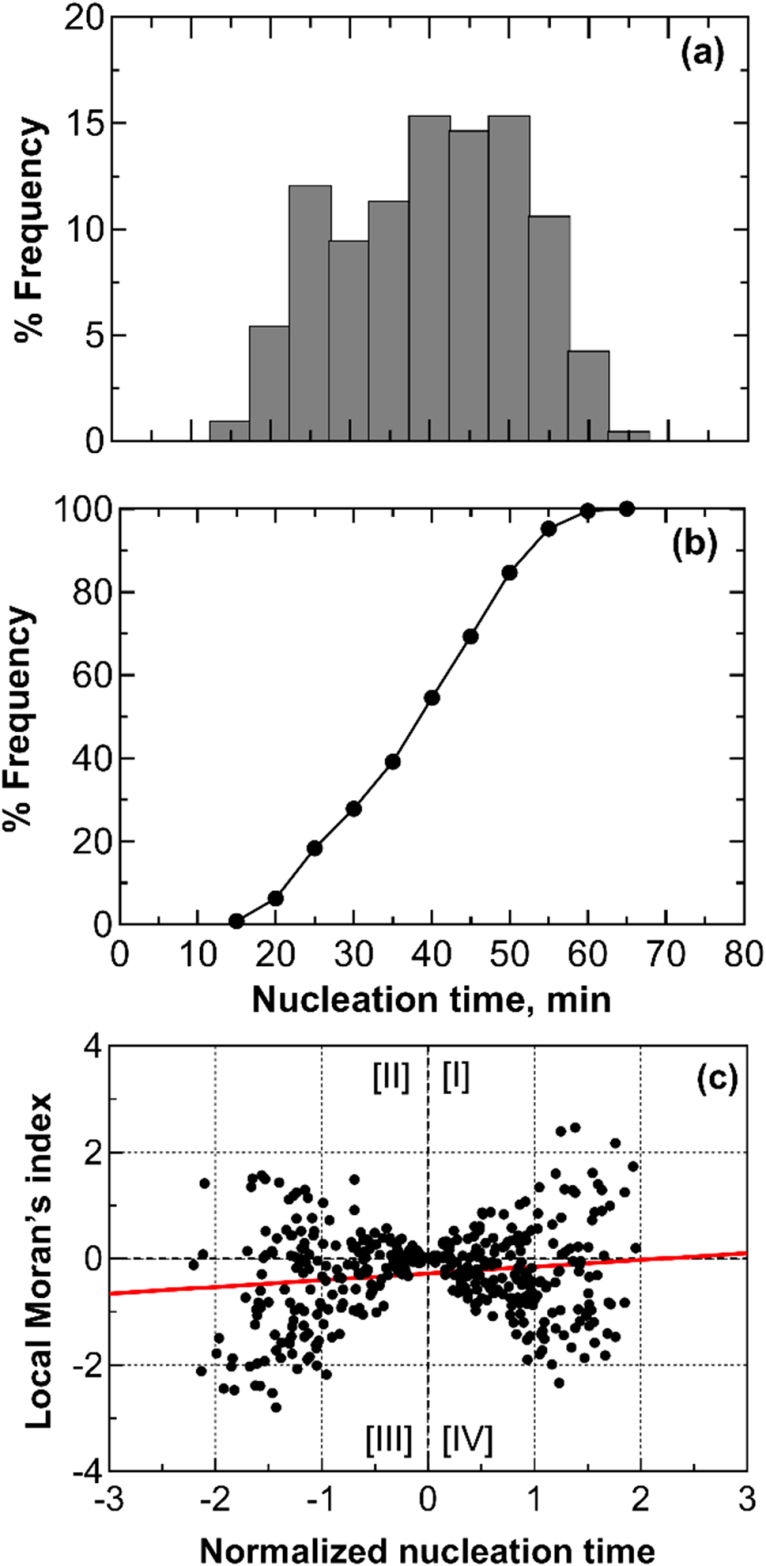
(**a**) Nucleation time distribution, (**b**) its cumulative function, and (**c**) scatterplot of standardized local Moran's indices for a batch of vials in direct contact with one another and the freeze-dryer shelf, but loaded on a stainless steel tray (configuration B). Data refer to three replicas involving 196 vials, each filled with 2 mL of a 5 wt% sucrose solution. The vials with thermocouples were omitted from the statistical analysis to avoid any potential interference that the sensor probes might have on the ice nucleation process.

This analysis was repeated loading 2R vials onto a stainless-steel tray while keeping the original batch layout (configuration B). This tray helps reduce potential temperature variations on the refrigerated shelf, ensuring consistent subcooling times. As can be seen in Fig. 4, nucleation times appeared randomly distributed. On average, it took about 40 min from the first vial nucleation to the last vial nucleation. Similar to the previous case, two peaks are observed at 25 and 45 min. Furthermore, the cumulative distribution function shows that within the first 25 min, 19% of the vials had nucleated, and between 25 and 45 min, 70% had initiated nucleation. After the second peak, 30% of the vials experienced delayed nucleation due to the heat released by vials nucleating within the second peak.

Figure 4c, showing the scatterplot of local Moran's indices against subcooling times, continues to confirm the presence of nucleation inhibition within the batch. The trendline reveals that most data points fell within the third and fourth quadrants, where the local Moran's indices are negative, reflecting the double-peak pattern in subcooling time distribution. Additionally, the global Moran's indices were between –0.05 and –0.14, confirming the presence of inhibition across all three replicas

Lastly, as can be seen in Table III, the metal tray has mitigated the presence of thermal gradients between vials.

### Packed Vials Suspended Over the Shelf

When vials are loaded directly on the shelf, the primary heat transfer mechanism is conduction between the bottom of the vial and the shelf. To mitigate this effect, vials were suspended above the refrigerated shelf, ensuring that heat primarily transfers through the walls of the vials. For this purpose, we utilized two plexiglass structures that enabled the simultaneous monitoring of the freezing process for 40 vials (20 vials for each structure). In this configuration, each vial was in contact with two other vials. The distance between the bottom of the vials and the refrigerated shelf was 20 mm, while the gap between parallel vials measured 18.3 mm.

**Fig. 5:**
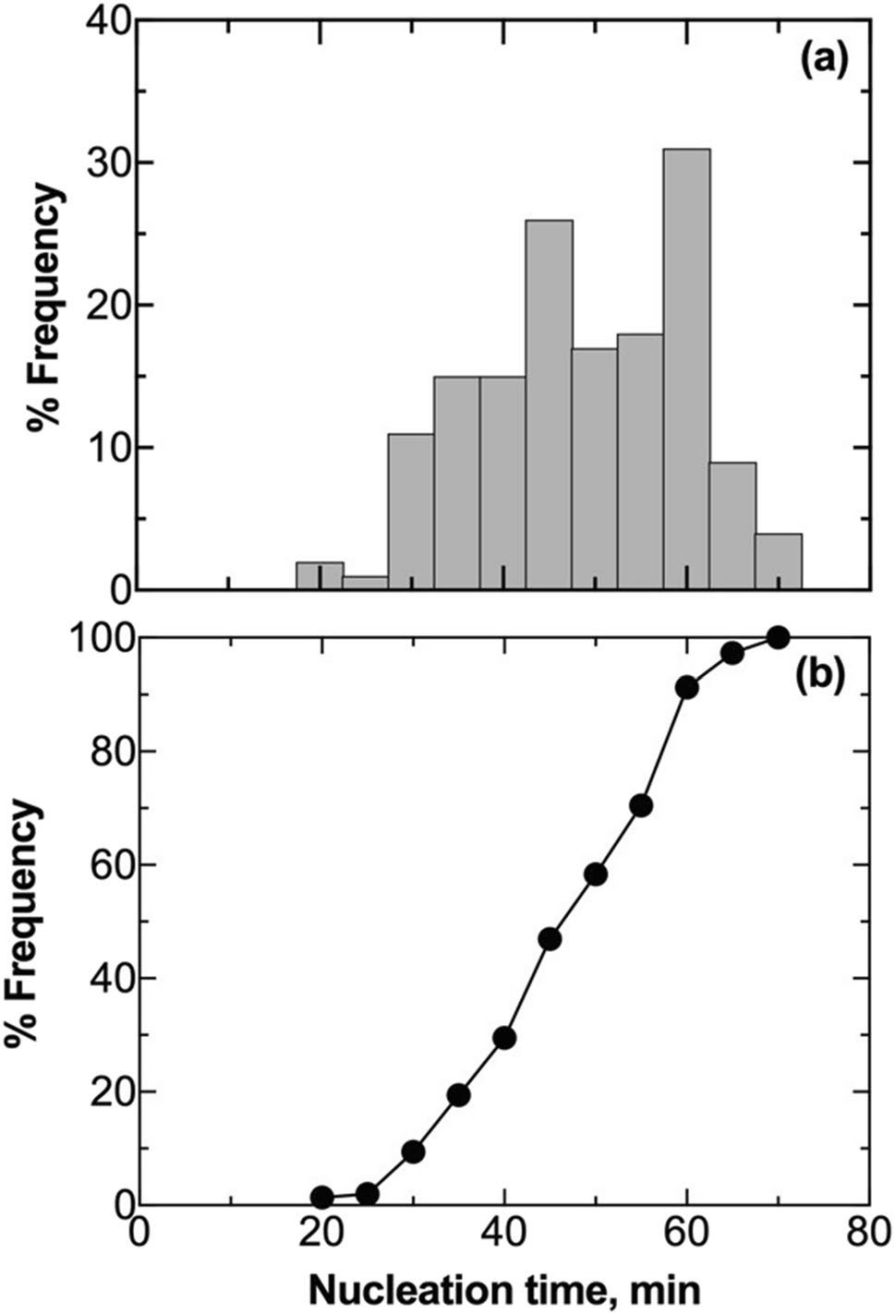
(**a**) Nucleation time distribution and (**b**) its cumulative function for a batch of vials in direct contact with one another and suspended over the shelves (configuration C). Data refer to three replicas involving 40 vials, each filled with 2 mL of a 5 wt% sucrose solution. The vials with thermocouples were omitted from the statistical analysis to avoid any potential interference that the sensor probes might have on the ice nucleation process.

Figure 5a shows the nucleation time distribution, with an average time between the nucleation of the first vial and the last vial of approximately 50 min, which was comparable to the case of packed vials directly loaded on the shelves. This distribution shows three distinct peaks; the first, at 30 min, marks the nucleation of 12% of the vials in the batch; the second peak at 45 min corresponds to 44% of the vials nucleating, and the final peak at 60 min refers to the nucleation of 91% of the vials. After the third peak, 9% of the vials experienced delayed nucleation due to the heat released by vials nucleating within the third peak. This result confirms that thermal interactions among adjacent vials primarily occur through heat transfer across the walls of the vials themselves.

### Vials Resting on the Shelf with Empty Vials in Between Them

**Fig. 6:**
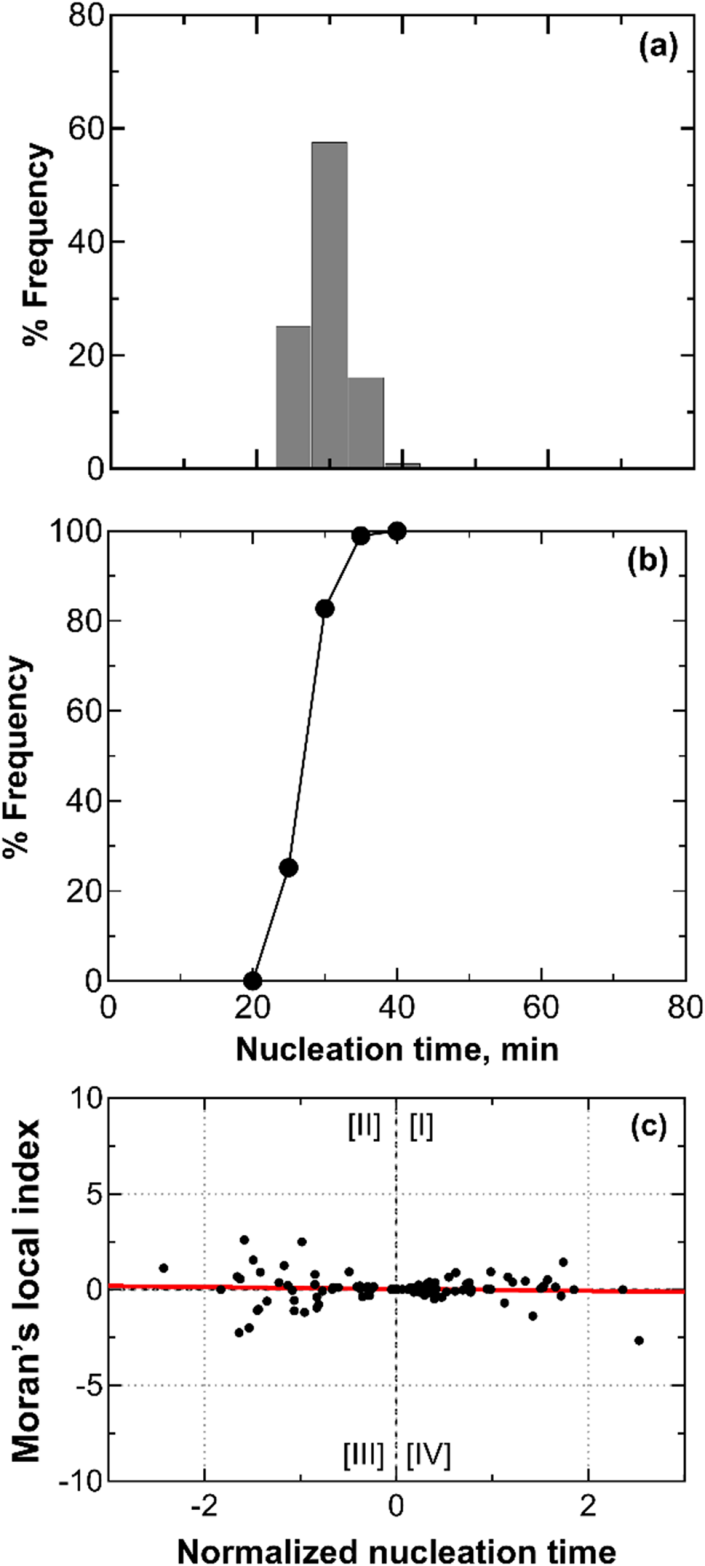
(**a**) Nucleation time distribution, (**b**) its cumulative function, and (**c**) scatterplot of standardized local Moran's indices for a batch of vials separated from one another by empty vials (configuration D). Data refer to three replicas involving 36 vials, each filled with 2 mL of a 5 wt% sucrose solution. The vials with thermocouples were omitted from the statistical analysis to avoid any potential interference that the sensor probes might have on the ice nucleation process.

In this section, each vial containing 2 mL of a 5 wt% sucrose solution was surrounded by 6 empty vials in order to mitigate the thermal interactions between the vials. Figure 6 shows that the nucleation times continued to exhibit a random distribution. However, the range of variation has been reduced by half compared to the previous tests. The characteristic double-peak pattern is no longer visible, confirming that the empty vials have dampened thermal interactions between adjacent vials.

**Fig. 7:**
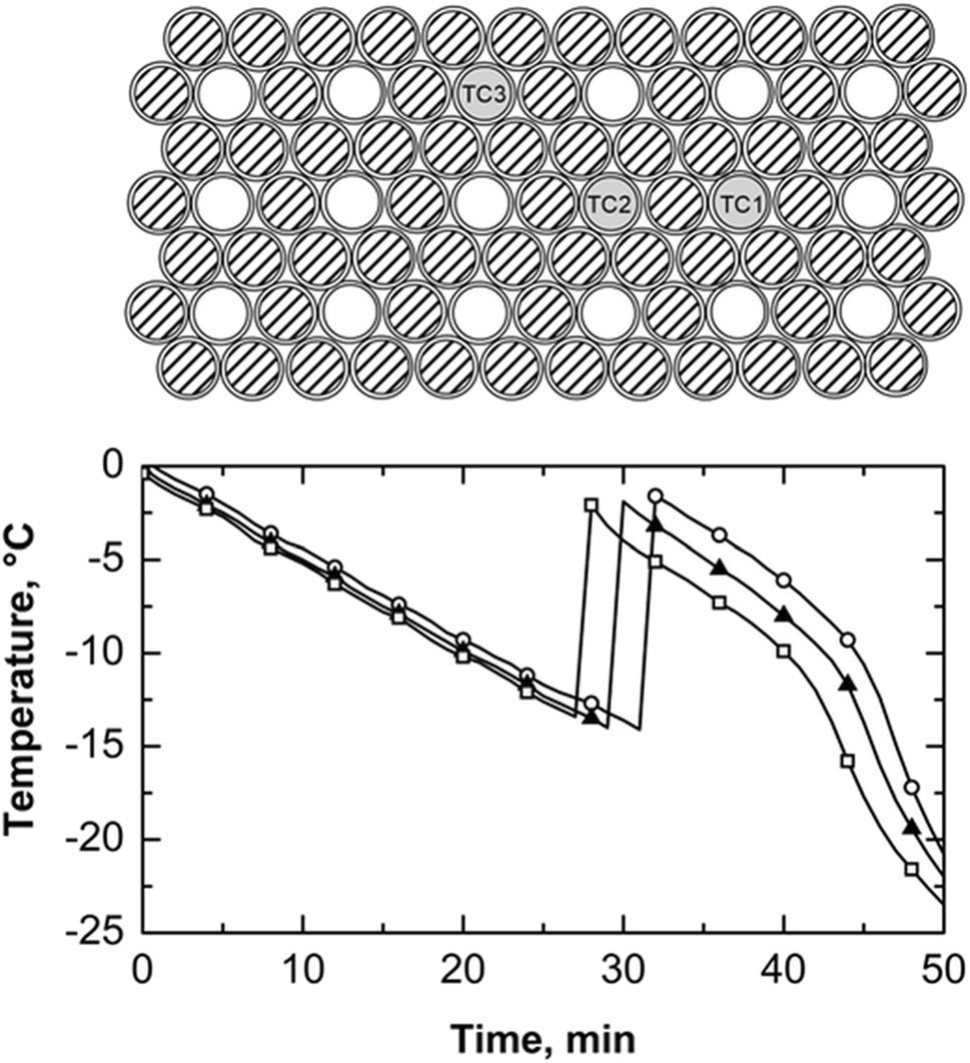
(top) Schematic illustrating the positioning of three thermocouples within the batch of vials separated from one another by empty vials, marked as dashed-filled (configuration D). (bottom) Temperature profiles of thermocouples TC1 (○), TC2 (▲), and TC3 (□).

The absence of thermal interactions is evident from the thermal profiles in Fig. 7, where even closely positioned vials (separated by one empty vial), such as vials #1 and #2, do not influence the thermal profile of the other in any instance. Furthermore, the nucleation times and temperatures of individual vials varied between repetitions, regardless of their shelf positions. For example, in the three repetitions, vial #3, positioned at the outer edge near the lyophilizer walls, nucleated at temperatures of –11.2 °C, –14.2 °C, and –13.4 °C, with subcooling times of 23, 30, and 28 min.

In Fig. 6c, a scatterplot of Moran's indices shows that most points cluster near zero on the x-axis, indicating null local Moran’s indices and minimal interactions between neighboring vials. The absence of inhibition is further supported by the global Moran's indices, $I=$ 0.03.

### Separated Vials Resting on the Shelf

The vials containing 2 mL of 5 wt% sucrose solution were spaced apart using cardboard supports. The decision to space the vials and employ a non-conductive material was made to observe how thermal interactions between vials could be affected. In the previous tests, heat released during solidification was transferred between adjacent samples through conduction via the glass vial walls. In this test, this conductive contribution was eliminated and replaced with convective heat transfer through the gas (air) present between the vials.

**Fig. 8:**
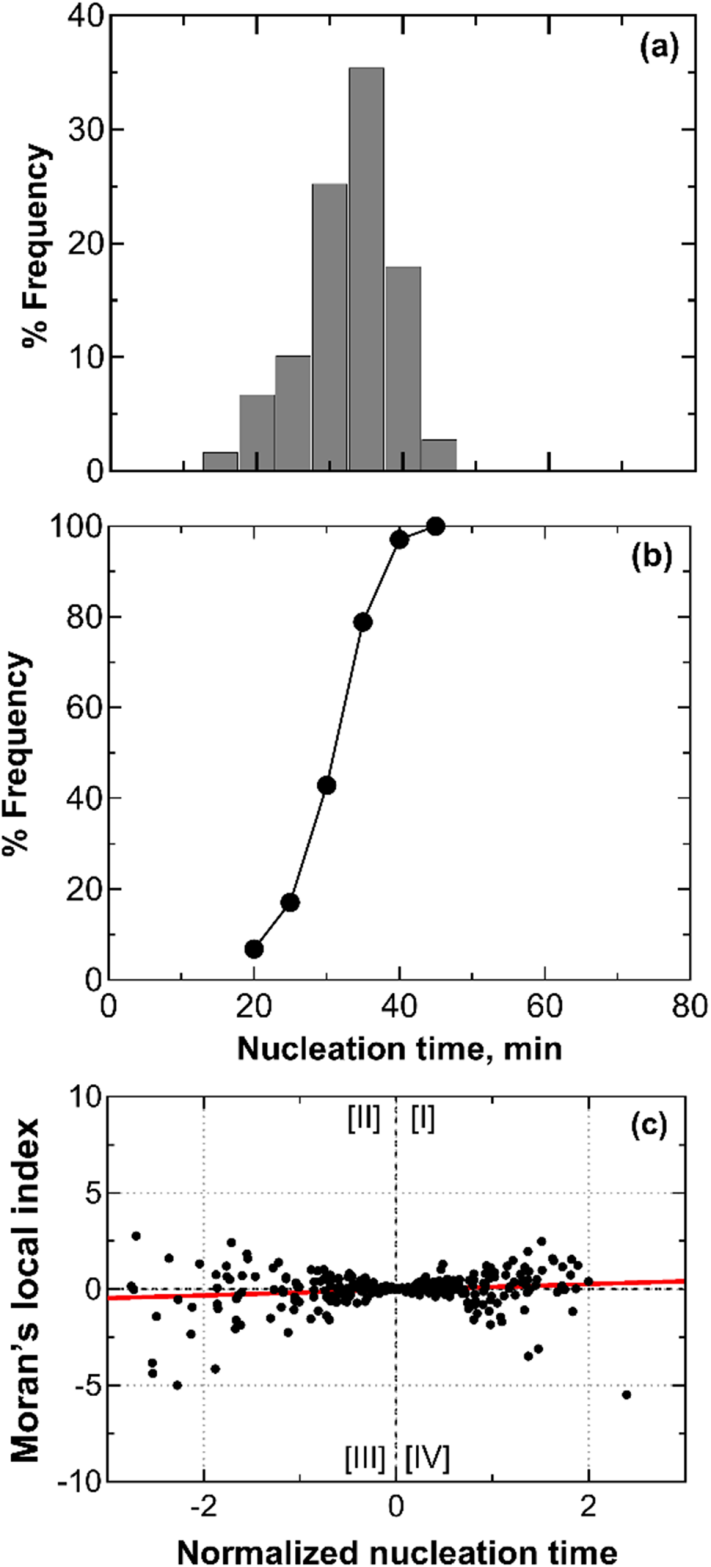
(**a**) Nucleation time distribution, (**b**) its cumulative function, and (**c**) scatterplot of standardized local Moran's indices for a batch of vials separated by one another through a thermally insulating material (configuration E). Data refer to three replicas involving 100 vials, each filled with 2 mL of a 5 wt% sucrose solution. The vials with thermocouples were omitted from the statistical analysis to avoid any potential interference that the sensor probes might have on the ice nucleation process.

Figure 8a shows that the nucleation times follow a unimodal random distribution, with a peak at 35 min. The time span between the nucleation of the first vial and the last vial is approximately 30 min.

The cumulative distribution function of subcooling times, shown in Fig. 8b, indicates that 60% of the vials in the batch nucleated within a nucleation time ranging from 30 to 40 min. In comparison to the case of vials separated through empty vials, the time distribution here exhibited a 10-min increase in width. This result suggests that, even though the double-peak pattern is absent, interactions were still present in this loading configuration.

**Fig. 9:**
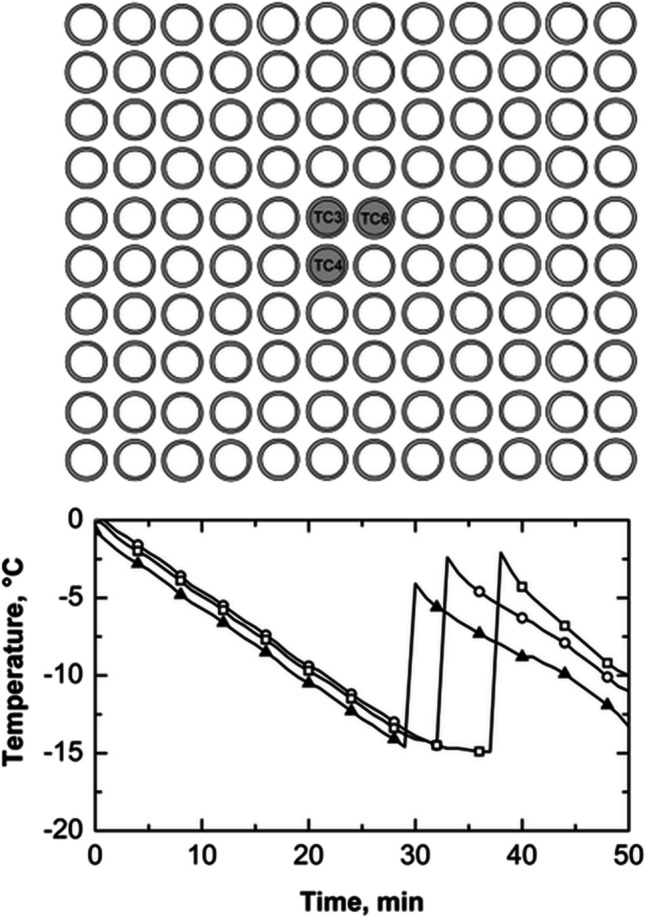
(top) Schematic illustrating the positioning of three thermocouples within the batch when vials are in direct contact with the freeze-dryer shelves and one another through a customized holder (configuration E). (bottom) Temperature profiles of thermocouples TC3 (□), TC4 (○), and TC6 (▲).

As can be seen in Fig. 9, the nucleation in vials #4 and #6 has affected the temperature behavior of the solution in vial #3. In contrast to the typical pattern observed in previous tests, where the temperature of the non-nucleated vial significantly increased as a consequence of the interaction despite ongoing cooling, the response of the system, i.e., vial #3, was different here. The slope of the temperature change before nucleation was less steep, and this change was due to the absorption of heat released during solidification from vials #4 and #6.

The global Moran's indices were in the range of –0.03 and –0.01. Although the trendline of the local Moran's indices shown in Fig. 8c suggests an absence of interactions, some vials exhibited negative indices with higher absolute values compared to those in Fig. 3c and Fig. 6c, which refer to the case of all vials filled with 5 wt% sucrose or with empty vials in between them.

### Vials Resting on the Shelf With Glycol-Filled Vials in Between Them

The vials containing sucrose were surrounded by vials containing ethylene glycol that do not nucleate in the range of temperature of interest. The final objective is to observe the perturbation in the thermal profiles of the solutions contained in the vials adjacent to the nucleation event.

**Fig. 10:**
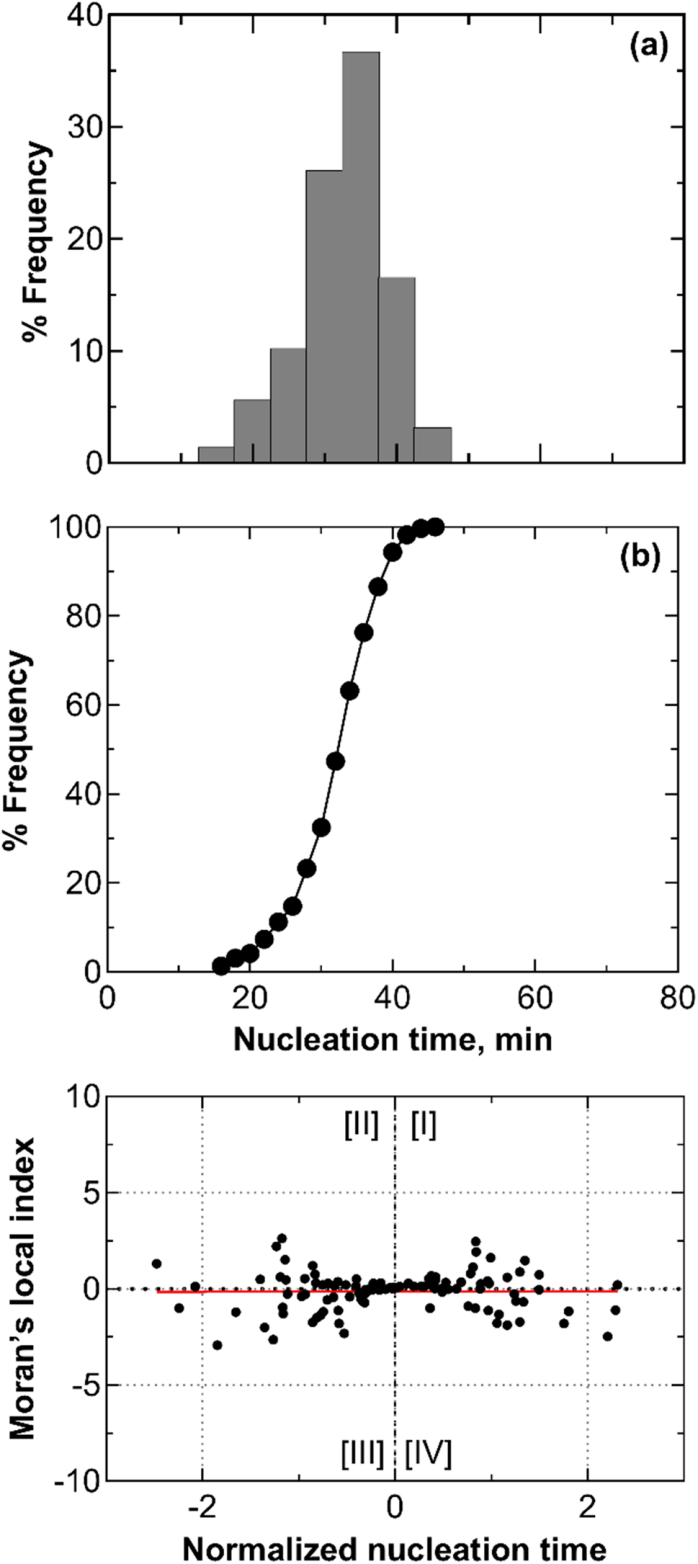
(**a**) Nucleation time distribution, (**b**) its cumulative function, and (**c**) a scatterplot of standardized local Moran's indices for a batch of vials separated by one another through glycol-filled vials (configuration F). Data refer to three replicas involving 36 vials, each filled with 2 mL of a 5 wt% sucrose solution. The vials with thermocouples were omitted from the statistical analysis to avoid any potential interference that the sensor probes might have on the ice nucleation process.

The nucleation times followed a unimodal random distribution. As depicted in Fig. 10, it was significantly narrower compared to all previous tests and showed a peak at 35 min. We can also observe that 76% of the vials in the batch nucleated within 35 min. These results suggest that glycol-filled vials effectively absorbed the heat released by neighboring vials undergoing nucleation. Due to their heat capacity, the glycol-filled vials did not transmit this heat to the sucrose-filled vials that had not yet nucleated. As a result, ice nucleation times in this setup were entirely randomly distributed. The global Moran’s index was between 0.05 and 0.00 range, confirming that the nucleation process is fully stochastic and glycol-filled vials dampen any thermal interactions between nucleating vials. The global Moran’s index fell within the range of 0.05 to 0.00, confirming that the nucleation process is entirely stochastic, and glycol-filled vials mitigate any thermal interactions between the nucleating vials.

## Conclusion

This research offers valuable insights into the thermal interactions in frozen pharmaceutical vials and their impact on nucleation times and inhibition. The findings of this study are of significance for the pharmaceutical industry, particularly in understanding freezing processes.

This study shows that the thermal interactions among vials during the freezing process are not to be underestimated. Vials in direct contact with neighboring vials and the refrigerated shelves experienced delayed nucleation, altering the expected nucleation temperature distribution. These delays can introduce variability in freezing behavior, affecting ice crystal size and potentially compromising product stability. This phenomenon is attributed to inhibition caused by heat transfer from early nucleating vials to those that are yet to nucleate. The use of a stainless-steel tray did not result in narrower nucleation time distributions, even if it has reduced thermal gradients within the batch of vials, suggesting that heat transfer through the vial walls was the primary factor in thermal interactions among adjacent vials. This finding was confirmed by the test using suspended vials.

Furthermore, this work has investigated the impact of different loading configurations, highlighting that the use of spacers, i.e., empty vials, cardboards, can mitigate thermal interactions between adjacent vials, yielding a more predictable and consistent nucleation time distribution. Nevertheless, these configurations are here intended only for scientific purposes, rather than being an alternative to industrially adopted ones. However, it is worth noting that the use of nested rack systems is now adopted as an industrial practice for vial loading. While thermal interactions enlarge the range of ice nucleation times, this does not necessarily result in a wider distribution of nucleation temperatures. This work shows that if it is true that the nucleation of one vial can delay the nucleation of the neighboring vial, it is also true that the temperature at which the non-nucleated vial eventually nucleates can be similar, even if there is a delay in the process. Therefore, thermal interactions between nucleating vials, in combination with annealing or forced-nucleation techniques, could be beneficial for batch uniformity. Unfortunately, this hypothesis cannot be directly validated because it would require a precise and non-invasive temperature mapping of individual vials in the batch. For this purpose, thermo-cameras might be used instead of thermocouples, but they are not sufficiently accurate in a low-temperature system immersed in a warmer chamber, which acts as a radiant body.

## List of Symbols

$I$: global Moran’s index, –
${I}_{i}$: local Moran’s index of the *i*-th vial, –
IQR: interquartile range for the nucleation time, min
${m}_{2}$: the second-order moment of the normalized nucleation time vector, –
${N}_{v}$: number of vial, –
${t}_{0}$: time corresponding to equilibrium freezing temperature, min
${t}_{f}$: time corresponding to the completion of freezing, min
${t}_{in}$: time corresponding to the onset of the cooling ramp, min
${t}_{n}$: nucleation time of individual vials, min
$\mathbf{y}$: centered nucleation time vector, –
$$\mathbf{W}$$: neighbor matrix, –
$\mathbf{z}$: normalized nucleation time vector, –
$\mathbf{Z}$: normalized and centered local Moran’s index vector, –

## Subscripts/superscripts

$$empty$$: empty
$$gly$$: ethylene glycol
$$s$$: sucrose
$$tot$$: total

## Greek letters

$\mu$: mean value of the nucleation time distribution, min
$\sigma$: standard deviation of the nucleation time distribution, min
${\sigma }_{I}$: standard deviation for the local Moran’s indices, –

## Abbreviations

TC: Thermocouple

## References

[1] Privalov PL. Cold denaturation of proteins. Crit Rev Biochem Mol Biol. 1990;25:281–305. 2225910 10.3109/10409239009090612

[2] Graziano G, Catanzano F, Riccio A, Barone G. A reassessment of the molecular origin of cold denaturation. J Biochem. 1997;122:395–401. 9378719 10.1093/oxfordjournals.jbchem.a021766

[3] Lopez CF, Darst RK, Rossky PJ. Mechanistic elements of protein cold denaturation. J Phys Chem B. 2008;112:5961–7. 18181599 10.1021/jp075928t

[4] Strambini GB, Gabellieri E. Proteins in frozen solutions: evidence of ice-induced partial unfolding. Biophys J. 1996;70:971–6. 8789114 10.1016/S0006-3495(96)79640-6 PMC1224997

[5] Arsiccio A, McCarty J, Pisano R, Shea J-E. Heightened cold-denaturation of proteins at the ice-water interface. J Am Chem Soc. 2020;142:5722–30. 32122128 10.1021/jacs.9b13454

[6] Arsiccio A, Pisano R. The ice-water interface and protein stability: a review. J Pharm Sci. 2020;109:2116–30. 32240686 10.1016/j.xphs.2020.03.022

[7] Arsiccio A, Giorsello P, Marenco L, Pisano R. Considerations on protein stability during freezing and its impact on the freeze-drying cycle: a design space approach. J Pharm Sci. 2020;109:464–75. 31647953 10.1016/j.xphs.2019.10.022

[8] Jiang S, Nail SL. Effect of process conditions on recovery of protein activity after freezing and freeze-drying. Eur J Pharm Biopharm. 1998;45:249–57. 9653629 10.1016/s0939-6411(98)00007-1

[9] Bhatnagar BS, Bogner RH, Pikal MJ. Protein stability during freezing: separation of stresses and mechanisms of protein stabilization. Pharm Dev Technol. 2007;12:505–23. 17963151 10.1080/10837450701481157

[10] Authelin J-R, Rodrigues MA, Tchessalov S, Singh SK, McCoy T, Wang S, Shalaev E. Freezing of biologicals revisited: scale, stability, excipients, and degradation stresses. J Pharm Sci. 2020;109:44–61. 31705870 10.1016/j.xphs.2019.10.062

[11] AboulFotouh K, Cui Z, Williams RO. Next-generation COVID-19 vaccines should take efficiency of distribution into consideration. AAPS PharmSciTech. 2021;22:126. 33835300 10.1208/s12249-021-01974-3 PMC8034273

[12] Kasper JC, Friess WF. The freezing step in lyophilisation: physico-chemical fundamentals, freezing methods and consequences on process performance and quality attributes of biopharmaceuticals. Eur J Pharm Biopharm. 2011;78:248–63. 21426937 10.1016/j.ejpb.2011.03.010

[13] Capozzi LC, Pisano R. Looking inside the ‘black box’: freeze engineering to ensure the quality of freeze-dried biopharmaceuticals. Eur J Pharm Biopharm. 2018;129:58–65. 29787801 10.1016/j.ejpb.2018.05.020

[14] Searles JA, Carpenter JF, Randolph TW. Annealing to optimize the primary drying rate, reduce freezing-induced drying rate heterogeneity, and determine T_g_’ in pharmaceutical lyophilization. J Pharm Sci. 2001;90:872–87. 11458336 10.1002/jps.1040

[15] Kubota N. Random distribution active site model for ice nucleation in water droplets. Cryst Eng Comm. 2019;21:3810–21.

[16] Deck L-T, Ochsenbein DR, Mazzotti M. Stochastic shelf-scale modeling framework for the freezing stage in freeze-drying processes. Int J Pharm. 2022;613:121276. 10.1016/j.ijpharm.2021.121276 34767908

[17] Deck L-T, Ochsenbein DR, Mazzotti M. Stochastic ice nucleation governs the freezing process of biopharmaceuticals in vials. Int J Pharm. 2022;625:122051. 10.1016/j.ijpharm.2022.122051 35907555

[18] Isenrich FN, Shardt N, Rösch M, Nette J, Stavrakis S, Marcolli C, et al. The microfluidic ice nuclei counter Zürich (MINCZ): a platform for homogeneous and heterogeneous ice nucleation. Atmos Meas Tech. 2022;15:5367–81.

[19] Stratta L, Arsiccio A, Pisano R. Effect of diffusion kinetics on the ice nucleation temperature distribution. Sci Reports. 2022;12:16334. 10.1038/s41598-022-20797-1 PMC9522862 36175610

[20] Chang BS, Kendrick BS, Carpenter JF. Surface-induced denaturation of proteins during freezing and its inhibition by surfactants. J Pharm Sci. 1996;85:1325–30. 8961147 10.1021/js960080y

[21] Murase N, Franks F. Salt precipitation during the freeze-concentration of phosphate buffer solutions. Biophys Chem. 1989;34:293–300. 2611352 10.1016/0301-4622(89)80066-3

[22] Bluemel O, Anuschek M, Buecheler JW, Hoelzl G, Bechtold-Peters K, Friess W. The effect of mAb and excipient cryoconcentration on long-term frozen storage stability. Part 1: higher molecular weight species and subvisible particle formation. Int J Pharm. 2022;4:100108. 10.1016/j.ijpx.2021.100108 PMC8724966 35024603

[23] Bluemel O, Buecheler JW, Hauptmann A, Hoelzl G, Bechtold-Peters K, Friess W. The effect of mAb and excipient cryoconcentration on long-term frozen storage stability. Part 2: aggregate formation and oxidation. Int J Pharm. 2022;4:100109. 10.1016/j.ijpx.2021.100109 PMC8724956 35024604

[24] Bald WB. On crystal size and cooling rate. J Microsc. 1986;143:89–102. 3531522 10.1111/j.1365-2818.1986.tb02767.x

[25] Arsiccio A, Marenco L, Pisano R. A model-based approach for the rational design of the freeze-thawing of a protein-based formulation. Pharm Dev Technol. 2020;25:823–31. 32367756 10.1080/10837450.2020.1743719

[26] Searles JA, Carpenter JF, Randolph TW. The ice nucleation temperature determines the primary drying rate of lyophilisation for samples frozen on a temperature-controlled shelf. J Pharm Sci. 2001;90:860–71. 11458335 10.1002/jps.1039

[27] Pisano R, Artusio F, Adami M, Barresi AA, Fissore D, Frare MC, et al. Freeze-drying of pharmaceuticals in vials nested in a rack system. Part I: freezing behaviour Pharmaceutics. 2023;15:635. 36839958 10.3390/pharmaceutics15020635 PMC9960346

[28] Pisano R. Alternative methods of controlling nucleation in freeze drying. In: Ward KR, Matejtschuk P, editors. Lyophilization of Pharmaceuticals and Biologicals: New Technologies and Approaches. 1st ed. New York, NY, USA: Springer; 2019. p. 79–111.

[29] Pisano R, Arsiccio A, Nakagawa K, Barresi AA. Tuning, measurement and prediction of the impact of freezing on product morphology: A step toward improved design of freeze-drying cycles. Drying Technol. 2019;37:579–99.

[30] Arsiccio A, Barresi AA, Pisano R. Prediction of ice crystal size distribution after freezing of pharmaceutical solutions. Cryst Growth Des. 2017;17:4573–81.

[31] Pisano R, Capozzi LC. Prediction of product morphology of lyophilised drugs in the case of Vacuum-Induced Surface Freezing. Chem Eng Res Des. 2017;125:119–29.

[32] Colucci D, Fissore D, Barresi AA, Braatz RD. A new mathematical model for monitoring the temporal evolution of the ice crystal size distribution during freezing in pharmaceutical solutions. Eur J Pharm Biopharm. 2020;148:148–59. 31953190 10.1016/j.ejpb.2020.01.004

[33] Buceta JP, Tréléa IC, Scutellà B, Bourlès E, Fonseca F, Passot S. Heat transfer during freeze-drying using a high-throughput vial system in view of process scale-up to serum vials. J Pharm Sci. 2021;110:1323–36. 33275993 10.1016/j.xphs.2020.11.029

[34] Matejčíková A, Tichý E, Rajniak P. Experimental investigation of inhomogeneities of primary drying during lyophilisation: Impact of the vials packing density. J Drug Deliv Sci Technol. 2022;74: 103550.

[35] Palmkron SB, Gustavsson L, Wahlgren M, Bergensthal B, Fureby AM. Temperature and heat transfer control during freeze drying. Effect of vial holders and influence of pressure. Pharm Res. 2022;39:2597–606. 35925479 10.1007/s11095-022-03353-4 PMC9556401

[36] Ehlers S, Schroeder R, Friess W. Trouble with the neighbor during freeze-drying: rivalry about energy. J Pharm Sci. 2021;110:1219–26. 33069707 10.1016/j.xphs.2020.10.024

[37] Maggioni GM, Mazzotti M. Modelling the stochastic behaviour of primary nucleation. Faraday Discuss. 2015;179:359–82. 25877048 10.1039/c4fd00255e

[38] Anselin L. Local indicators of spatial association - LISA. Geogr Anal. 1995;27:93–115.

[39] Chen Y. New approaches for calculating Moran’s index of spatial autocorrelation. PLoS One. 2013;8:1–14. 10.1371/journal.pone.0068336 PMC3709922 23874592

